# Supplementary material for: Medication Adherence among Allogeneic Haematopoietic Stem Cell Transplant Recipients: A Systematic Review
Source: Cancers (Basel). 2023 Apr 25;15(9):2452. doi: 10.3390/cancers15092452 (PMC10177142; doi:10.3390/cancers15092452)
Supplement: Supplementary file 1 [file cancers-15-02452-s001.zip › cancers-2351915-supplementary.pdf]

Table S1. The 2020 PRISMA Checklist [34].

Section and Topic	Item #	Checklist item	Location where item is reported
**TITLE**			
Title	1	Identify the report as a systematic review.	Title and first paragraph of Materials and Methods
**ABSTRACT**			
Abstract	2	See the PRISMA 2020 for Abstracts checklist (Table S1.2)	Abstract
**INTRODUCTION**			
Rationale	3	Describe the rationale for the review in the context of existing knowledge.	Introduction
Objectives	4	Provide an explicit statement of the objective(s) or question(s) the review addresses.	Last lines of Introduction
**METHODS**			
Eligibility criteria	5	Specify the inclusion and exclusion criteria for the review and how studies were grouped for the syntheses.	Selection criteria
Information sources	6	Specify all databases, registers, websites, organisations, reference lists and other sources searched or consulted to identify studies. Specify the date when each source was last searched or consulted.	Study selection and search strategy
Search strategy	7	Present the full search strategies for all databases, registers and websites, including any filters and limits used.	Table S2
Selection process	8	Specify the methods used to decide whether a study met the inclusion criteria of the review, including how many reviewers screened each record and each report retrieved, whether they worked independently, and if applicable, details of automation tools used in the process.	Study selection and search strategy
Data collection process	9	Specify the methods used to collect data from reports, including how many reviewers collected data from each report, whether they worked independently, any processes for obtaining or confirming data from study investigators, and if applicable, details of automation tools used in the process.	Data extraction
Data items	10a	List and define all outcomes for which data were sought. Specify whether all results that were compatible with each outcome domain in each study were sought (e.g. for all measures, time points, analyses), and if not, the methods used to decide which results to collect.	Data extraction
	10b	List and define all other variables for which data were sought (e.g. participant and intervention characteristics, funding sources). Describe any assumptions made about any missing or unclear information.	Data extraction
Study risk of bias assessment	11	Specify the methods used to assess risk of bias in the included studies, including details of the tool(s) used, how many reviewers assessed each study and whether they worked independently, and if applicable, details of automation tools used in the process.	Quality assessment and confidence in cumulative evidence
Effect measures	12	Specify for each outcome the effect measure(s) (e.g. risk ratio, mean difference) used in the synthesis or presentation of results.	Data synthesis and analysis

Section and Topic	Item #	Checklist item	Location where item is reported
Synthesis methods	13a	Describe the processes used to decide which studies were eligible for each synthesis (e.g. tabulating the study intervention characteristics and comparing against the planned groups for each synthesis (item #5)).	Selection criteria
	13b	Describe any methods required to prepare the data for presentation or synthesis, such as handling of missing summary statistics, or data conversions.	Data synthesis and analysis
	13c	Describe any methods used to tabulate or visually display results of individual studies and syntheses.	Data synthesis and analysis
	13d	Describe any methods used to synthesize results and provide a rationale for the choice(s). If meta-analysis was performed, describe the model(s), method(s) to identify the presence and extent of statistical heterogeneity, and software package(s) used.	NA
	13e	Describe any methods used to explore possible causes of heterogeneity among study results (e.g. subgroup analysis, meta-regression).	NA
	13f	Describe any sensitivity analyses conducted to assess robustness of the synthesized results.	NA
Reporting bias assessment	14	Describe any methods used to assess risk of bias due to missing results in a synthesis (arising from reporting biases).	Quality assessment and confidence in cumulative evidence
Certainty assessment	15	Describe any methods used to assess certainty (or confidence) in the body of evidence for an outcome.	Quality assessment and confidence in cumulative evidence
**RESULTS**			
Study selection	16a	Describe the results of the search and selection process, from the number of records identified in the search to the number of studies included in the review, ideally using a flow diagram.	Figure 1
	16b	Cite studies that might appear to meet the inclusion criteria, but which were excluded, and explain why they were excluded.	Figure 1 and Table S3
Study characteristics	17	Cite each included study and present its characteristics.	Study main characteristics and Table S5
Risk of bias in studies	18	Present assessments of risk of bias for each included study.	Table S4
Results of individual studies	19	For all outcomes, present, for each study: (a) summary statistics for each group (where appropriate) and (b) an effect estimate and its precision (e.g. confidence/credible interval), ideally using structured tables or plots.	Oral MA: assessment tools and rates, Factors affecting MA/MNA, Figure 2, Interventions to improve MA, Clinical

Section and Topic	Item #	Checklist item	Location where item is reported
			outcomes of MNA, Table 1, Table 2 and Table 3
Results of syntheses	20a	For each synthesis, briefly summarise the characteristics and risk of bias among contributing studies.	Quality assessment and confidence in cumulative
	20b	Present results of all statistical syntheses conducted. If meta-analysis was done, present for each the summary estimate and its precision (e.g. confidence/credible interval) and measures of statistical heterogeneity. If comparing groups, describe the direction of the effect.	NA
	20c	Present results of all investigations of possible causes of heterogeneity among study results.	NA
	20d	Present results of all sensitivity analyses conducted to assess the robustness of the synthesized results.	NA
Reporting biases	21	Present assessments of risk of bias due to missing results (arising from reporting biases) for each synthesis assessed.	NA
Certainty of evidence	22	Present assessments of certainty (or confidence) in the body of evidence for each outcome assessed.	Quality assessment and confidence in cumulative evidence
**DISCUSSION**			
Discussion	23a	Provide a general interpretation of the results in the context of other evidence.	Discussion
	23b	Discuss any limitations of the evidence included in the review.	Limitations
	23c	Discuss any limitations of the review processes used.	Limitations
	23d	Discuss implications of the results for practice, policy, and future research.	Conclusions
**OTHER INFORMATION**			
Registration and protocol	24a	Provide registration information for the review, including register name and registration number, or state that the review was not registered.	First lines of Materials and Methods
	24b	Indicate where the review protocol can be accessed, or state that a protocol was not prepared.	First lines of Materials and Methods
	24c	Describe and explain any amendments to information provided at registration or in the protocol.	Quality assessment and confidence in cumulative evidence
Support	25	Describe sources of financial or non-financial support for the review, and the role of the funders or sponsors in the review.	Funding
Competing interests	26	Declare any competing interests of review authors.	Conflicts of Interest

Section and Topic	Item #	Checklist item	Location where item is reported
Availability of data, code and other materials	27	Report which of the following are publicly available and where they can be found: template data collection forms; data extracted from included studies; data used for all analyses; analytic code; any other materials used in the review.	Data Availability Statement

NA: not applicable.

Table S2. The 2020 PRISMA for Abstract checklist [34].

Section and topic	Item #	Checklist item
**TITLE**		
Title	1	Identify the report as a systematic review.
**BACKGROUND**		
Objectives	2	Provide an explicit statement of the main objective(s) or question(s) the review addresses.
**METHODS**		
Eligibility criteria	3	Specify the inclusion and exclusion criteria for the review.
Information sources	4	Specify the information sources (e.g. databases, registers) used to identify studies and the date when each was last searched.
Risk of bias	5	Specify the methods used to assess risk of bias in the included studies.
Synthesis of results	6	Specify the methods used to present and synthetize results.
**RESULTS**		
Included studies	7	Give the total number of included studies and participants and summarize relevant characteristics of studies.
Synthesis of results	8	Present results for main outcomes, preferably indicating the number of included studies and participants for each. If meta-analysis was done, report the summary estimate and confidence/credible interval. If comparing groups, indicate the direction of the effect (i.e. which group is favoured).
**DISCUSSION**		
Limitations of evidence	9	Provide a brief summary of the limitations of the evidence included in the review (e.g. study risk of bias, inconsistency and imprecision).
Interpretation	10	Provide a general interpretation of the results and important implications.
**OTHER**		
Funding	11	Specify the primary source of funding for the review.

Registration	12	Provide the register name and registration number.

Table S3. Search strategies for the six selected databases.

String number	Researched outcome(s)	Search strings	Results
		**PubMed (n = 3121):** duplicates (n = 1669), remaining (n = 1452)	
#1	MA/MNA	("Hematopoietic Stem Cell Transplantation"[Mesh] OR "Transplantation, Homologous"[Mesh] OR stem cell transplant* OR bone marrow transplant* OR haematopoietic stem cell transplant* OR hematopoietic stem cell transplant* OR allogeneic transplant* OR haploidentical transplant*) AND ("Medication Adherence"[Mesh] OR medication adherence OR adherence medication OR medication nonadherence OR medication non-adherence OR medication non adherence OR Drug Adherence OR Medication Persistence OR medication noncompliance OR medication compliance OR medication non-compliance OR compliance OR adherence) AND (pharmaceutic* OR pharmaceutical preparation* OR pharmaceutical product* OR drug* OR "Pharmaceutical Preparations"[Mesh] OR pharmaceutical regime* OR regime* OR "oral therapy" OR pill* OR tablet* OR capsule OR "Immunosuppressive Agents"[Mesh] OR immunosuppressant* OR nonimmunosuppressant* OR non-immunosuppressant*)	1432
#2	MNA and risk factors	(#1 OR pre-transplant*) AND ("Risk Factors"[Mesh] OR risk factor* OR social risk factor* OR health correlate* OR risk score* OR risk factor score* OR predictor* OR screening OR "screening tool" OR psychosocial assessment* OR evaluation* OR psychosocial* OR facilitate* OR barrier*)	479
#3	Interventions increasing MA	#1 AND ("Psychosocial Intervention"[Mesh] OR intervention* OR psychological intervention* OR "Patient Education as Topic"[Mesh] OR "patient education" OR education OR consultation* OR counselling* OR implementation OR self-medication)	209
#4	MA/MNA and GvHD	#1 AND ("Graft vs Host Disease"[Mesh] OR graft-versus-host disease* OR graft-versus-host OR graft versus host disease* OR graft-vs-host disease*)	88
#5	MA/MNA and infections	#1 AND ("Infections"[Mesh] OR viral infection* OR bacterial infection* OR fungal infection* OR sepsis*)	177
#6	MA/MNA and hospital readmission	#1 AND ("Patient Readmission"[Mesh] OR patient readmission* OR unplanned readmission OR hospital readmission* OR rehospitalization OR intensive care unit admission* OR intensive care unit OR emergency room visit*)	24
#7	MA/MNA and mortality	#1 AND ("Survival"[Mesh] OR "Mortality"[Mesh] OR survival OR survivor* OR mortality OR death OR mortality rate* OR progression-free survival)	501
#8	MA/MNA and disease relapse	#1 AND ("Recurrence"[Mesh] OR recurrence* OR recrudescence* OR relapse* OR disease relapse*)	100
		**CINAHL (n = 289):** duplicates (n = 176), remaining (n = 113)	

#1	MA/MNA	(stem cell transplant* OR bone marrow transplant* OR haematopoietic stem cell transplant* OR hematopoietic stem cell transplant* OR allogeneic transplant* OR haploidentical transplant* OR (MH "Bone Marrow Transplantation") OR (MH "Bone Marrow Transplantation, Allogeneic")) AND ((MH "Medication Compliance") OR (MH "Compliance with Medical Regimen (Saba CCC)") OR (MH "Compliance with Medication Regimen (Saba CCC)" OR medication adherence OR adherence medication OR medication nonadherence OR medication non-adherence OR medication non adherence OR Drug Adherence OR Medication Persistence OR medication noncompliance OR medication compliance OR medication non-compliance OR compliance OR adherence) AND (pharmaceutic* OR pharmaceutical preparation* OR pharmaceutical product* OR drug* OR pharmaceutical regime* OR regime* OR “oral therapy” OR pill* OR tablet* OR capsule OR Immunosuppressive Agent* OR immunosuppressant* OR nonimmunosuppressant* OR non-immunosuppressant* OR (MH "Immunosuppressive Agents") OR (MH "Immunosuppression"))	110
#2	MNA and risk factors	(#1 OR pre-transplant*) AND (risk factor* OR social risk factor* OR health correlate* OR risk score* OR risk factor score* OR predictor* OR screening OR “screening tool” OR psychosocial assessment* OR evaluation* OR psychosocial* OR facilitate* OR barrier* OR (MH "Risk Factors") OR (MH "Social Factors"))	60
#3	Interventions increasing MA	#1 AND (Psychosocial Intervention* OR intervention* OR psychological intervention* OR patient education OR education* OR consultation* OR counselling* OR implementation OR self-medication OR (MH "Psychosocial Intervention") OR (MH "Nursing Interventions") OR (MH "Intervention Trials") OR (MH "Patient Education") OR (MH "Counseling"))	34
#4	MA/MNA and GvHD	#1 AND (Graft vs Host Disease OR graft-versus-host disease* OR graft-versus-host OR graft versus host disease* OR graft-vs-host disease* OR (MH "Graft Versus Host Disease"))	11
#5	MA/MNA and infections	#1 AND (Infection* OR viral infection* OR bacterial infection* OR fungal infection* OR sepsis* OR (MH "Sepsis") OR (MH "Infection"))	24
#6	MA/MNA and hospital readmission	#1 AND (patient readmission* OR unplanned readmission* OR hospital readmission* OR rehospitalization OR intensive care unit admission* OR intensive care unit OR emergency room visit* OR (MH "Readmission") OR (MH "Intensive Care Units"))	5
#7	MA/MNA and mortality	#1 AND (survival OR survivor* OR mortality OR death OR mortality rate* OR progression-free survival OR (MH "Survival") OR (MH "Mortality") OR (MH "Death"))	35
#8	MA/MNA and disease relapse	#1 AND (recurrence* OR recrudescence* OR relapse* OR disease relapse* OR (MH "Recurrence"))	11
		**Cochrane Library (n = 761):** duplicates (n = 520), remaining (n = 241)	
#1	MA/MNA	(stem cell transplant* OR bone marrow transplant* OR haematopoietic stem cell transplant* OR hematopoietic stem cell transplant* OR allogeneic transplant* OR haploidentical transplant*) AND (medication adherence OR adherence medication OR medication nonadherence OR medication non-adherence OR medication non adherence OR Drug Adherence OR Medication Persistence OR medication noncompliance OR medication compliance OR medication non-compliance OR compliance OR adherence) AND (pharmaceutic* OR pharmaceutical preparation* OR pharmaceutical product* OR drug* OR pharmaceutical regime* OR regime* OR “oral therapy” OR pill* OR tablet* OR capsule OR Immunosuppressive Agent* OR immunosuppressant* OR nonimmunosuppressant* OR non-immunosuppressant*)	244

#2	MNA and risk factors	(#1 OR pre-transplant*) AND (risk factor* OR social risk factor* OR health correlate* OR risk score* OR risk factor score* OR predictor* OR screening OR "screening tool" OR psychosocial assessment* OR evaluation* OR psychosocial* OR facilitate* OR barrier*)	113
#3	Interventions increasing MA	#1 AND (Psychosocial Intervention* OR intervention* OR psychological intervention* OR patient education OR education* OR consultation* OR counselling* OR implementation OR self-medication)	98
#4	MA/MNA and GvHD	#1 AND (Graft vs Host Disease OR graft-versus-host disease* OR graft-versus-host OR graft versus host disease* OR graft-vs-host disease*)	37
#5	MA/MNA and infections	#1 AND (Infection* OR viral infection* OR bacterial infection* OR fungal infection* OR sepsis*)	53
#6	MA/MNA and hospital readmission	#1 AND (patient readmission* OR unplanned readmission* OR hospital readmission* OR rehospitalization OR intensive care unit admission* OR intensive care unit OR emergency room visit*)	6
#7	MA/MNA and mortality	#1 AND (survival OR survivor* OR mortality OR death OR mortality rate* OR progression-free survival)	145
#8	MA/MNA and disease relapse	#1 AND (recurrence* OR recrudescence* OR relapse* OR disease relapse*)	79
**PsycINFO (n = 2315):** duplicates (n = 1757), remaining (n = 558)			
#1	MA/MNA	(stem cell transplant* OR bone marrow transplant* OR haematopoietic stem cell transplant* OR hematopoietic stem cell transplant* OR allogeneic transplant* OR haploidentical transplant*) AND (medication adherence OR adherence medication OR medication nonadherence OR medication non-adherence OR medication non adherence OR Drug Adherence OR Medication Persistence OR medication noncompliance OR medication compliance OR medication non-compliance OR compliance OR adherence) AND (pharmaceutic* OR pharmaceutical preparation* OR pharmaceutical product* OR drug* OR pharmaceutical regime* OR regime* OR oral therapy OR pill* OR tablet* OR capsule OR Immunosuppressive Agent* OR immunosuppressant* OR nonimmunosuppressant* OR non-immunosuppressant*)	522
#2	MNA and risk factors	(#1 OR pre-transplant*) AND (risk factor* OR social risk factor* OR health correlate* OR risk score* OR risk factor score* OR predictor* OR screening OR screening tool OR psychosocial assessment* OR evaluation* OR psychosocial* OR facilitate* OR barrier*)	540
#3	Interventions increasing MA	#1 AND (Psychosocial Intervention* OR intervention* OR psychological intervention* OR patient education OR education* OR consultation* OR counselling* OR implementation OR self-medication)	434
#4	MA/MNA and GvHD	#1 AND (Graft vs Host Disease OR graft-versus-host disease* OR graft-versus-host OR graft versus host disease* OR graft-vs-host disease*)	20
#5	MA/MNA and infections	#1 AND (Infection* OR viral infection* OR bacterial infection* OR fungal infection* OR sepsis*)	153
#6	MA/MNA and hospital readmission	#1 AND (patient readmission* OR unplanned readmission* OR hospital readmission* OR rehospitalization OR intensive care unit admission* OR intensive care unit OR emergency room visit*)	59
#7	MA/MNA and mortality	#1 AND (survival OR survivor* OR mortality OR death OR mortality rate* OR progression-free survival)	426
#8	MA/MNA and disease relapse	#1 AND (recurrence* OR recrudescence* OR relapse* OR disease relapse*)	161
**SCOPUS (n = 718):** duplicates (n = 579), remaining (n = 139)			

#1	MA/MNA	(stem cell transplant* OR bone marrow transplant* OR haematopoietic stem cell transplant* OR hematopoietic stem cell transplant* OR allogeneic transplant* OR haploidentical transplant*) AND (medication adherence OR adherence medication OR medication nonadherence OR medication non-adherence OR medication non adherence OR Drug Adherence OR Medication Persistence OR medication noncompliance OR medication compliance OR medication non-compliance OR compliance OR adherence) AND (pharmaceutic* OR pharmaceutical preparation* OR pharmaceutical product* OR drug* OR pharmaceutical regime* OR regime* OR “oral therapy” OR pill* OR tablet* OR capsule OR Immunosuppressive Agent* OR immunosuppressant* OR nonimmunosuppressant* OR non-immunosuppressant*)	138
#2	MNA and risk factors	(#1 OR pre-transplant*) AND (risk factor* OR social risk factor* OR health correlate* OR risk score* OR risk factor score* OR predictor* OR screening OR “screening tool” OR psychosocial assessment* OR evaluation* OR psychosocial* OR facilitate* OR barrier*)	105
#3	Interventions increasing MA	#1 AND (Psychosocial Intervention* OR intervention* OR psychological intervention* OR patient education OR education* OR consultation* OR counselling* OR implementation OR self-medication)	38
#4	MA/MNA and GvHD	#1 AND (Graft vs Host Disease OR graft-versus-host disease* OR graft-versus-host OR graft versus host disease* OR graft-vs-host disease*)	37
#5	MA/MNA and infections	#1 AND (Infection* OR viral infection* OR bacterial infection* OR fungal infection* OR sepsis*)	107
#6	MA/MNA and hospital readmission	#1 AND (patient readmission* OR unplanned readmission* OR hospital readmission* OR rehospitalization OR intensive care unit admission* OR intensive care unit OR emergency room visit*)	3
#7	MA/MNA and mortality	#1 AND (survival OR survivor* OR mortality OR death OR mortality rate* OR progression-free survival)	98
#8	MA/MNA and disease relapse	#1 AND (recurrence* OR recrudescence* OR relapse* OR disease relapse*)	56
		**EMBASE (n = 1793):** duplicates (n = 908), remaining (n = 885)	
#1	MA/MNA	(stem cell transplant* OR bone marrow transplant* OR haematopoietic stem cell transplant* OR hematopoietic stem cell transplant* OR allogeneic transplant* OR haploidentical transplant*) AND (medication adherence OR adherence medication OR medication nonadherence OR medication non-adherence OR medication non adherence OR Drug Adherence OR Medication Persistence OR medication noncompliance OR medication compliance OR medication non-compliance OR compliance OR adherence) AND (pharmaceutic* OR pharmaceutical preparation* OR pharmaceutical product* OR drug* OR pharmaceutical regime* OR regime* OR “oral therapy” OR pill* OR tablet* OR capsule OR Immunosuppressive Agent* OR immunosuppressant* OR nonimmunosuppressant* OR non-immunosuppressant*) ('stem cell transplant*':ti,ab,kw OR 'bone marrow transplant*':ti,ab,kw OR 'haematopoietic stem cell transplant*':ti,ab,kw OR 'hematopoietic stem cell transplant*':ti,ab,kw OR 'allogeneic transplant*':ti,ab,kw OR 'haploidentical transplant*':ti,ab,kw) AND ('medication adherence':ti,ab,kw OR 'adherence medication':ti,ab,kw OR 'medication nonadherence':ti,ab,kw OR 'medication non-adherence':ti,ab,kw OR 'medication non adherence':ti,ab,kw OR 'drug adherence':ti,ab,kw OR 'medication persistence':ti,ab,kw OR 'medication noncompliance':ti,ab,kw OR 'medication compliance':ti,ab,kw OR 'medication non-compliance':ti,ab,kw OR compliance:ti,ab,kw OR adherence:ti,ab,kw) AND (pharmaceutic*:ti,ab,kw OR 'pharmaceutical preparation*':ti,ab,kw OR 'pharmaceutical product*':ti,ab,kw OR drug*:ti,ab,kw OR 'pharmaceutical regime*':ti,ab,kw OR regime*:ti,ab,kw OR 'oral therapy':ti,ab,kw OR pill*:ti,ab,kw OR tablet*:ti,ab,kw OR capsule:ti,ab,kw OR 'immunosuppressive agent*':ti,ab,kw OR immunosuppressant*:ti,ab,kw OR nonimmunosuppressant*:ti,ab,kw OR 'non immunosuppressant*':ti,ab,kw)	594

#2	MNA and risk factors	(#1 OR pre-transplant*) AND (risk factor* OR social risk factor* OR health correlate* OR risk score* OR risk factor score* OR predictor* OR screening OR “screening tool” OR psychosocial assessment* OR evaluation* OR psychosocial* OR facilitate* OR barrier*)<br>('risk factor*':ti,ab,kw OR 'social risk factor*':ti,ab,kw OR 'health correlate*':ti,ab,kw OR 'risk score*':ti,ab,kw OR 'risk factor score*':ti,ab,kw OR predictor*:ti,ab,kw OR screening:ti,ab,kw OR 'screening tool':ti,ab,kw OR 'psychosocial assessment*':ti,ab,kw OR evaluation*:ti,ab,kw OR psychosocial*:ti,ab,kw OR facilitate*:ti,ab,kw OR barrier*:ti,ab,kw) AND ('pre transplant*':ti,ab,kw OR 'stem cell transplant*':ti,ab,kw OR 'bone marrow transplant*':ti,ab,kw OR 'haematopoietic stem cell transplant*':ti,ab,kw OR 'hematopoietic stem cell transplant*':ti,ab,kw OR 'allogeneic transplant*':ti,ab,kw OR 'haploidentical transplant*':ti,ab,kw) AND ('medication adherence':ti,ab,kw OR 'adherence medication':ti,ab,kw OR 'medication nonadherence':ti,ab,kw OR 'medication non-adherence':ti,ab,kw OR 'medication non adherence':ti,ab,kw OR 'drug adherence':ti,ab,kw OR 'medication persistence':ti,ab,kw OR 'medication noncompliance':ti,ab,kw OR 'medication compliance':ti,ab,kw OR 'medication non-compliance':ti,ab,kw OR compliance:ti,ab,kw OR adherence:ti,ab,kw) AND (pharmaceutic*:ti,ab,kw OR 'pharmaceutical preparation*':ti,ab,kw OR 'pharmaceutical product*':ti,ab,kw OR drug*:ti,ab,kw OR 'pharmaceutical regime*':ti,ab,kw OR regime*:ti,ab,kw OR 'oral therapy':ti,ab,kw OR pill*:ti,ab,kw OR tablet*:ti,ab,kw OR capsule:ti,ab,kw OR 'immunosuppressive agent*':ti,ab,kw OR immunosuppressant*:ti,ab,kw OR nonimmunosuppressant*:ti,ab,kw OR 'non immunosuppressant*':ti,ab,kw)	230
#3	Interventions increasing MA	#1 AND (Psychosocial Intervention* OR intervention* OR psychological intervention* OR patient education OR education* OR consultation* OR counselling* OR implementation OR self-medication)<br>('psychosocial intervention*':ti,ab,kw OR intervention*:ti,ab,kw OR 'psychological intervention*':ti,ab,kw OR 'patient education':ti,ab,kw OR education*:ti,ab,kw OR consultation*:ti,ab,kw OR counselling*:ti,ab,kw OR implementation:ti,ab,kw OR 'self medication':ti,ab,kw) AND ('stem cell transplant*':ti,ab,kw OR 'bone marrow transplant*':ti,ab,kw OR 'haematopoietic stem cell transplant*':ti,ab,kw OR 'hematopoietic stem cell transplant*':ti,ab,kw OR 'allogeneic transplant*':ti,ab,kw OR 'haploidentical transplant*':ti,ab,kw) AND ('medication adherence':ti,ab,kw OR 'adherence medication':ti,ab,kw OR 'medication nonadherence':ti,ab,kw OR 'medication non-adherence':ti,ab,kw OR 'medication non adherence':ti,ab,kw OR 'drug adherence':ti,ab,kw OR 'medication persistence':ti,ab,kw OR 'medication noncompliance':ti,ab,kw OR 'medication compliance':ti,ab,kw OR 'medication non-compliance':ti,ab,kw OR compliance:ti,ab,kw OR adherence:ti,ab,kw) AND (pharmaceutic*:ti,ab,kw OR 'pharmaceutical preparation*':ti,ab,kw OR 'pharmaceutical product*':ti,ab,kw OR drug*:ti,ab,kw OR 'pharmaceutical regime*':ti,ab,kw OR regime*:ti,ab,kw OR 'oral therapy':ti,ab,kw OR pill*:ti,ab,kw OR tablet*:ti,ab,kw OR capsule:ti,ab,kw OR 'immunosuppressive agent*':ti,ab,kw OR immunosuppressant*:ti,ab,kw OR nonimmunosuppressant*:ti,ab,kw OR 'non immunosuppressant*':ti,ab,kw)	170
#4	MA/MNA and GvHD	#1 AND (Graft vs Host Disease OR graft-versus-host disease* OR graft-versus-host OR graft versus host disease* OR graft-vs-host disease*)<br>(('stem cell'/exp OR 'stem cell' OR (('stem'/exp OR stem) AND ('cell'/exp OR cell) AND transplant*) OR 'bone marrow'/exp OR 'bone marrow' OR (('bone'/exp OR bone) AND ('marrow'/exp OR marrow) AND transplant*) OR 'haematopoietic stem cell'/exp OR 'haematopoietic stem cell' OR (haematopoietic AND ('stem'/exp OR stem) AND ('cell'/exp OR cell) AND transplant*) OR 'hematopoietic stem cell'/exp OR 'hematopoietic stem cell' OR (hematopoietic AND ('stem'/exp OR stem) AND ('cell'/exp OR cell) AND transplant*) OR allogeneic) AND transplant* OR haploidentical) AND transplant* AND ('medication adherence'/exp OR 'medication adherence' OR (('medication'/exp OR medication)	135

		AND ('adherence'/exp OR adherence)) OR 'adherence medication' OR (('adherence'/exp OR adherence) AND ('medication'/exp OR medication)) OR 'medication nonadherence' OR (('medication'/exp OR medication) AND nonadherence) OR 'medication non-adherence' OR (('medication'/exp OR medication) AND 'non adherence') OR 'medication non adherence' OR (('medication'/exp OR medication) AND non AND ('adherence'/exp OR adherence)) OR 'drug adherence'/exp OR 'drug adherence' OR (('drug'/exp OR drug) AND ('adherence'/exp OR adherence)) OR 'medication persistence'/exp OR 'medication persistence' OR (('medication'/exp OR medication) AND ('persistence'/exp OR persistence)) OR 'medication noncompliance' OR (('medication'/exp OR medication) AND noncompliance) OR 'medication compliance'/exp OR 'medication compliance' OR (('medication'/exp OR medication) AND ('compliance'/exp OR compliance)) OR 'medication non-compliance' OR (('medication'/exp OR medication) AND 'non compliance') OR 'compliance'/exp OR compliance OR 'adherence'/exp OR adherence) AND (((((pharmaceutic* OR pharmaceutical) AND preparation* OR pharmaceutical) AND product* OR drug* OR pharmaceutical) AND regime* OR regime* OR 'oral therapy' OR pill* OR tablet* OR 'capsule'/exp OR capsule OR immunosuppressive) AND agent* OR immunosuppressant* OR nonimmunosuppressant* OR 'non immunosuppressant*') AND (('graft vs host disease'/exp OR 'graft vs host disease' OR (('graft'/exp OR graft) AND vs AND ('host'/exp OR host) AND ('disease'/exp OR disease)) OR 'graft versus host') AND disease* OR 'graft versus host' OR (('graft'/exp OR graft) AND versus AND ('host'/exp OR host) AND disease*) OR 'graft vs host') AND disease*	
#5	MA/MNA and infections	#1 AND (Infection* OR viral infection* OR bacterial infection* OR fungal infection* OR sepsis*) (('stem cell'/exp OR 'stem cell' OR (('stem'/exp OR stem) AND ('cell'/exp OR cell) AND transplant*) OR 'bone marrow'/exp OR 'bone marrow' OR (('bone'/exp OR bone) AND ('marrow'/exp OR marrow) AND transplant*) OR 'haematopoietic stem cell'/exp OR 'haematopoietic stem cell' OR (haematopoietic AND ('stem'/exp OR stem) AND ('cell'/exp OR cell) AND transplant*) OR 'hematopoietic stem cell'/exp OR 'hematopoietic stem cell' OR (hematopoietic AND ('stem'/exp OR stem) AND ('cell'/exp OR cell) AND transplant*) OR allogeneic) AND transplant* OR haploidentical) AND transplant* AND ('medication adherence'/exp OR 'medication adherence' OR (('medication'/exp OR medication) AND ('adherence'/exp OR adherence)) OR 'adherence medication' OR (('adherence'/exp OR adherence) AND ('medication'/exp OR medication)) OR 'medication nonadherence' OR (('medication'/exp OR medication) AND nonadherence) OR 'medication non-adherence' OR (('medication'/exp OR medication) AND 'non adherence') OR 'medication non adherence' OR (('medication'/exp OR medication) AND non AND ('adherence'/exp OR adherence)) OR 'drug adherence'/exp OR 'drug adherence' OR (('drug'/exp OR drug) AND ('adherence'/exp OR adherence)) OR 'medication persistence'/exp OR 'medication persistence' OR (('medication'/exp OR medication) AND ('persistence'/exp OR persistence)) OR 'medication noncompliance' OR (('medication'/exp OR medication) AND noncompliance) OR 'medication compliance'/exp OR 'medication compliance' OR (('medication'/exp OR medication) AND ('compliance'/exp OR compliance)) OR 'medication non-compliance' OR (('medication'/exp OR medication) AND 'non compliance') OR 'compliance'/exp OR compliance OR 'adherence'/exp OR adherence) AND (((((pharmaceutic* OR pharmaceutical) AND preparation* OR pharmaceutical) AND product* OR drug* OR pharmaceutical) AND regime* OR regime* OR 'oral therapy' OR pill* OR tablet* OR 'capsule'/exp OR capsule OR immunosuppressive) AND agent* OR immunosuppressant* OR nonimmunosuppressant* OR 'non immunosuppressant*') AND ((((infection* OR viral) AND infection* OR bacterial) AND infection* OR fungal) AND infection* OR sepsis*)	226

#6	MA/MNA and hospital readmission	#1 AND (patient readmission* OR unplanned readmission* OR hospital readmission* OR rehospitalization OR intensive care unit admission* OR intensive care unit OR emergency room visit*)<br>('stem cell transplant*':ti,ab,kw OR 'bone marrow transplant*':ti,ab,kw OR 'haematopoietic stem cell transplant*':ti,ab,kw OR 'hematopoietic stem cell transplant*':ti,ab,kw OR 'allogeneic transplant*':ti,ab,kw OR 'haploidentical transplant*':ti,ab,kw) AND ('medication adherence':ti,ab,kw OR 'adherence medication':ti,ab,kw OR 'medication nonadherence':ti,ab,kw OR 'medication non-adherence':ti,ab,kw OR 'medication non adherence':ti,ab,kw OR 'drug adherence':ti,ab,kw OR 'medication persistence':ti,ab,kw OR 'medication noncompliance':ti,ab,kw OR 'medication compliance':ti,ab,kw OR 'medication non-compliance':ti,ab,kw OR compliance:ti,ab,kw OR adherence:ti,ab,kw) AND (pharmaceutic*:ti,ab,kw OR 'pharmaceutical preparation*':ti,ab,kw OR 'pharmaceutical product*':ti,ab,kw OR drug*:ti,ab,kw OR 'pharmaceutical regime*':ti,ab,kw OR regime*:ti,ab,kw OR 'oral therapy':ti,ab,kw OR pill*:ti,ab,kw OR tablet*:ti,ab,kw OR capsule:ti,ab,kw OR 'immunosuppressive agent*':ti,ab,kw OR immunosuppressant*:ti,ab,kw OR nonimmunosuppressant*:ti,ab,kw OR 'non immunosuppressant*':ti,ab,kw) AND ('patient readmission*':ti,ab,kw OR 'unplanned readmission*':ti,ab,kw OR 'hospital readmission*':ti,ab,kw OR rehospitalization:ti,ab,kw OR 'intensive care unit admission*':ti,ab,kw OR 'intensive care unit':ti,ab,kw OR 'emergency room visit*':ti,ab,kw)	15
#7	MA/MNA and mortality	#1 AND (survival OR survivor* OR mortality OR death OR mortality rate* OR progression-free survival)<br>('stem cell transplant*':ti,ab,kw OR 'bone marrow transplant*':ti,ab,kw OR 'haematopoietic stem cell transplant*':ti,ab,kw OR 'hematopoietic stem cell transplant*':ti,ab,kw OR 'allogeneic transplant*':ti,ab,kw OR 'haploidentical transplant*':ti,ab,kw) AND ('medication adherence':ti,ab,kw OR 'adherence medication':ti,ab,kw OR 'medication nonadherence':ti,ab,kw OR 'medication non-adherence':ti,ab,kw OR 'medication non adherence':ti,ab,kw OR 'drug adherence':ti,ab,kw OR 'medication persistence':ti,ab,kw OR 'medication noncompliance':ti,ab,kw OR 'medication compliance':ti,ab,kw OR 'medication non-compliance':ti,ab,kw OR compliance:ti,ab,kw OR adherence:ti,ab,kw) AND (pharmaceutic*:ti,ab,kw OR 'pharmaceutical preparation*':ti,ab,kw OR 'pharmaceutical product*':ti,ab,kw OR drug*:ti,ab,kw OR 'pharmaceutical regime*':ti,ab,kw OR regime*:ti,ab,kw OR 'oral therapy':ti,ab,kw OR pill*:ti,ab,kw OR tablet*:ti,ab,kw OR capsule:ti,ab,kw OR 'immunosuppressive agent*':ti,ab,kw OR immunosuppressant*:ti,ab,kw OR nonimmunosuppressant*:ti,ab,kw OR 'non immunosuppressant*':ti,ab,kw) AND (survival:ti,ab,kw OR survivor*:ti,ab,kw OR mortality:ti,ab,kw OR death:ti,ab,kw OR 'mortality rate*':ti,ab,kw OR 'progression-free survival':ti,ab,kw)	265
#8	MA/MNA and disease relapse	#1 AND (recurrence* OR recrudescence* OR relapse* OR disease relapse*)<br>(('stem cell'/exp OR 'stem cell' OR (('stem'/exp OR stem) AND ('cell'/exp OR cell) AND transplant*) OR 'bone marrow'/exp OR 'bone marrow' OR (('bone'/exp OR bone) AND ('marrow'/exp OR marrow) AND transplant*) OR 'haematopoietic stem cell'/exp OR 'haematopoietic stem cell' OR (haematopoietic AND ('stem'/exp OR stem) AND ('cell'/exp OR cell) AND transplant*) OR 'hematopoietic stem cell'/exp OR 'hematopoietic stem cell' OR (hematopoietic AND ('stem'/exp OR stem) AND ('cell'/exp OR cell) AND transplant*) OR allogeneic) AND transplant* OR haploidentical) AND transplant* AND ('medication adherence'/exp OR 'medication adherence' OR (('medication'/exp OR medication) AND ('adherence'/exp OR adherence)) OR 'adherence medication' OR (('adherence'/exp OR adherence) AND ('medication'/exp OR medication)) OR 'medication nonadherence' OR (('medication'/exp OR medication) AND nonadherence) OR 'medication non-adherence' OR (('medication'/exp OR medication) AND 'non adherence') OR 'medication non adherence' OR (('medication'/exp OR medication) AND non AND ('adherence'/exp OR adherence)) OR 'drug adherence'/exp OR 'drug adherence' OR (('drug'/exp OR drug) AND ('adherence'/exp OR adherence)) OR 'medication persistence'/exp OR 'medication persistence'	143

	OR (('medication'/exp OR medication) AND ('persistence'/exp OR persistence)) OR 'medication noncompliance' OR (('medication'/exp OR medication) AND noncompliance) OR 'medication compliance'/exp OR 'medication compliance' OR (('medication'/exp OR medication) AND ('compliance'/exp OR compliance)) OR 'medication non-compliance' OR (('medication'/exp OR medication) AND 'non compliance') OR 'compliance'/exp OR compliance OR 'adherence'/exp OR adherence) AND (((((pharmaceutic* OR pharmaceutical) AND preparation* OR pharmaceutical) AND product* OR drug* OR pharmaceutical) AND regime* OR regime* OR 'oral therapy' OR pill* OR tablet* OR 'capsule'/exp OR capsule OR immunosuppressive) AND agent* OR immunosuppressant* OR nonimmunosuppressant* OR 'non immunosuppressant*') AND (recurrence* OR recrudescence* OR relapse* OR 'disease'/exp OR disease) AND relapse*

CINAHL: Cumulative Index of Nursing and Allied Health; EMBASE: Excerpta Medica database; GvHD: graft-versus-host disease; MA: medication adherence; MNA: medication non-adherence.

Table S4. Reasons for the exclusion after agreement among the three researchers.

First author, year	Reason(s)
Harashima et al., 2019 [36]	Investigated pre-HSCT MA with the PACT scale was not correlated with post-HSCT adherence
Kirsch et al., 2014 [37]	Mean follow-up time was not in line with the inclusion criteria (> 4 years)
Saleh, 2002 [38]	It included autologous HSCT without showing MA data for autologous and allogeneic HSCT<br>It included paediatrics and the moment of HSCT

HSCT: haematopoietic stem cell transplantation; MA: medication adherence; PACT: Psychosocial Assessment of Candidates for Transplantation.

Table S5. Quality assessment of included studies: critical appraisal tools divided by study design.

Analytical cross-sectional studies [40]

	Belaiche et al. 2020 [5]	Gresch et al. 2017 [14]	Ice et al. 2020 [6]	Mishkin et al. 2019 [11]	Posluszny et al. 2018 [12]
**Item 1**. Were the criteria for inclusion in the sample clearly defined?	Y	Y	Y	Y	Y
**Item 2**. Were the study subjects and the setting described in detail?	Y	Y	Y	Y	Y
**Item 3**. Was the exposure measured in a valid and reliable way?	Y	Y	Y	Y	NA
**Item 4**. Were objective, standard criteria used for measurement of the condition?	Y	Y	Y	Y	NA
**Item 5**. Were confounding factors identified?	N	Y	Y	Y	N
**Item 6**. Were strategies to deal with confounding factors stated?	N	Y	Y	Y	N
**Item 7**. Were the outcomes measured in a valid and reliable way?	Y	Y	Y	N	Y
**Item 8**. Was appropriate statistical analysis used?	Y	Y	Y	Y	Y

Quasi-experimental studies (non-randomised experimental studies) [39]

	Charra et al. 2021 [7]	Chieng et al. 2013 [24]	Polito et al. 2021 [25]	Zanetti et al. 2022 [26]
**Item 1**. Is it clear in the study what is the 'cause' and what is the 'effect' (i.e. there is no confusion about which variable comes first)?	Y	Y	Y	Y
**Item 2**. Were the participants included in any comparisons similar?	Y	NA	Y	NA
**Item 3**. Were the participants included in any comparisons receiving similar treatment/care, other than the exposure or intervention of interest?	Y	NA	Y	NA
**Item 4**. Was there a control group?	Y*	NA	Y	NA
**Item 5**. Were there multiple measurements of the outcome both pre- and post-the intervention/exposure?	Y	Y	Y	Y
**Item 6**. Was follow up complete and if not, were differences between groups in terms of their follow up adequately described and analysed?	Y	NA	Y	Y
**Item 7**. Were the outcomes of participants included in any comparisons measured in the same way?	Y	NA	Y	NA
**Item 8**. Were outcomes measured in a reliable way?	Y	U	Y	Y
**Item 9.** Was appropriate statistical analysis used?	N	Y	Y	Y

**Studies reporting prevalence data [41]**			
	García-Basas et al. 2020 [8]	Lehrer et al. 2018 [32]	Hoodin 1993 [10]
**Item 1**. Was the sample frame appropriate to address the target population?	Y	Y	Y
**Item 2**. Were study participants sampled in an appropriate way?	Y	U	Y
**Item 3**. Was the sample size adequate?	Y	U	U
**Item 4**. Were the study subjects and the setting described in detail?	Y	Y	Y
**Item 5**. Was the data analysis conducted with sufficient coverage of the identified sample?	Y	Y	Y
**Item 6**. Were valid methods used for the identification of the condition?	Y	Y	N
**Item 7**. Was the condition measured in a standard, reliable way for all participants?	U	Y	Y
**Item 8**. Was there appropriate statistical analysis?	Y	Y	Y
**Item 9.** Was the response rate adequate, and if not, was the low response rate managed appropriately?	Y	Y	Y

**Cohort studies [40]**		
	Scherer et al. 2021 [15]	Posluszny et al. 2022 [9]
**Item 1**. Were the two groups similar and recruited from the same population?	NA	NA
**Item 2**. Were the exposures measured similarly to assign people to both exposed and unexposed groups?	NA	NA
**Item 3**. Was the exposure measured in a valid and reliable way?	Y	Y
**Item 4**. Were confounding factors identified?	N	Y
**Item 5**. Were strategies to deal with confounding factors stated?	N	Y
**Item 6**. Were the groups/participants free of the outcome at the start of the study (or at the moment of exposure)?	Y	Y
**Item 7**. Were the outcomes measured in a valid and reliable way?	Y	Y
**Item 8**. Was the follow up time reported and sufficient to be long enough for outcomes to occur?	Y	Y
**Item 9.** Was follow up complete, and if not, were the reasons to loss to follow up described and explored?	Y	Y

**Item 10.** Were strategies to address incomplete follow up utilised?	U	U
**Item 11.** Was appropriate statistical analysis used?	Y	Y

Y: yes; N: no; U: unclear; NA: not applicable.
*A historical control group was used.

Table S6. Characteristics of the included studies (n = 14).

First author,<br>year,<br>country	Purpose(s) of the study	Study design,<br>setting,<br>study period	Population:<br>N,<br>mean age ± SD (range),<br>prevailing gender (n, %),<br>race/ethnicity (n, %),<br>haematological disease(s) (n, %),<br>type of allogeneic HSCT (n, %),<br>time from HSCT	Framework used to identify MA
		**Cross-sectional studies**		
Belaiche et al.,<br>2021 [5],<br>France	1. To assess MA<br>2. To identify factors associated with MA	Cross-sectional multicenter survey<br>19 allo-HSCT French centres<br>September 2015-March 2016	N = 203 adults (39 children from 6 centres not included in the results)<br>51.3 ± 13.5 years<br>Male = 122 (50.7%)<br>NR<br>Leukaemia = 132 (58.7)<br>NR<br>Mean of 4.5 months	MA is composed by three phases (initiation, implementation, persistence) [18]
Gresch et al.,<br>2017 [14],<br>Switzerland	1. To describe the prevalence of different forms of MNA<br>2. To examine associations between MNA and selected correlates<br>3. To explore the association between immunosuppressive MNA and cGvHD	Secondary data analysis of the cross-sectional multicenter study PROVIVO [37]<br>University Hospitals of Basel and Zurich<br>November 2011-November 2012	N = 99<br>51.0 (20.3-71.8) years<br>Male = 61 (61.6%)<br>NR<br>AML, ALL = 49 (49.5%)<br>Lymphoma, CLL, MM = 27 (27.2%)<br>CML, MDS, MPS = 22 (22.2%)<br>MUD = 52 (52.5%)<br>HLA-identical = 44 (44.4%)<br>Haploidentical = 3 (3.0%)<br>Mean of 3.9 years	MNA is the deviation from the prescribed medication regiment sufficient to adversely influence the regimen's intended effect [18]
Ice et al.,<br>2020 [6],<br>USA	To describe the prevalence of MNA to immunosuppressant and nonimmunosuppressant medications	Cross-sectional study (electronic survey)<br>Outpatient Bone Marrow Transplant Clinic at Mayo Clinic Hospital<br>December 2014-Aprile 2015	N = 200<br>56.6 (20-73) years<br>Male = 121 (60.5%)<br>Caucasian = 192 (96.0%)<br>NR<br>MUD = 94 (47.0%)<br>HLA-identical = 92 (46.0%)<br>Haploidentical = 3 (1.5%)	NR

			At least 3 months until 2 years	
Mishkin et al., 2019 [11], USA	1. To determine whether SIPAT ratings based on pre-transplantation psychiatric evaluation predict nonadherence and medical outcomes in the HSCT population<br>2. To evaluate the risk of mortality and causes of death in high-SIPAT and nonadherent groups	Retrospective study<br>Large academic urban medical centre in New York<br>August 2014-December 2016	N = 85*<br>47 (18-74) years<br>Male = 56 (66.0%)<br>NR<br>NHL = 21 (24.7%)<br>AML = 16 (18.8%)<br>MM = 15 (17.6%)<br>MDS = 10 (11.7%)<br>HL = 10 (11.7%)<br>Other = 13 (15.3%)<br>Autologous = 43 (50.5%)<br>Allogeneic = 42 (50.0%)<br>MUD = 18 (42.8%)<br>HLA-identical = 15 (35.7%)<br>Haploidentical = 2 (4.8%)<br>1 year	NA as a binary variable, defined as occurrence of at least 1 life-threatening nonadherence event in the first 6 months post-HSCT.<br>NA includes the following categories: drug use, medications, hospitalization, diet, infection risk, appointments and examinations, psychiatric and physical therapy
Posluszny et al., 2018 [12], USA	To describe:<br>1. adherence levels across post-HSCT medical regimen tasks<br>2. how post-HSCT adherence levels differ across tasks and how dyads differ in the division of task responsibilities<br>To explore:<br>3. the contribution of dyadic factors to successful adherence in HSCT<br>4. the associations between task adherence and demographic, psychological, and clinical factors	Cross-sectional study<br>HSCT center of the University of Pittsburgh Medical Center-Hillman Cancer Center<br>18-month period	N = 42<br>N = 21 (50.0%) patients<br>N = 21 (50.0%) caregivers<br>50.8 ± 12.6 (24-71) years (patients)<br>52.5 ± 12.6 (23-75) years (caregivers)<br>Female = 12 (57.0%) (patients)<br>Female = 13 (62.0%) (caregivers)<br>European American = 20 (95.0%)<br>African American = 1 (5.0%)<br>AML = 15 (71.0%)<br>Other = 6 (29.0%)<br>MUD = 15 (71.0%)<br>HLA-identical = 6 (29.0%)<br>83.4 ± 30.9 (44-145) days	NR<br>Adherence includes the following categories: medication use, infection precautions, lifestyle, central venous access management, alcohol and tobacco consumption and clinic appointments
		**Quasi-experimental studies**		
Charra et al., 2021 [7], France	To evaluate the impact of a specialized clinical pharmacy program on:	Prospective open interventional study with a retrospective control group<br>HSCT unit of French university Hospital<br>Prospective cohort: August 2017-April 2018	N = 61<br>N = 26 (42.6%) intervention group<br>N = 35 (57.4%) control group<br>51.8 (29-66) years (intervention group)<br>50.3 (23-70) years (control group)	NR

	1. adherence to oral immunosuppression treatment after discharge from HSCT unit<br>2. aGvHD and infections	Retrospective cohort: year 2016	Male = 15 (57.7%) (intervention group)<br>Male = 22 (62.9%) (control group)<br>NR<br>Acute leukaemia = 15 (57.7%), other = 11 (42.3%) (intervention group)<br>Acute leukaemia = 22 (62.9%), other = 13 (37.1%) (control group)<br>Related donor = 9 (34.6%), MUD = 17 (65.4%) (intervention group)<br>Related donor = 21 (60.0%), MUD = 14 (40.0%) (intervention group)<br>100 days	
Chieng et al., 2013 [24], Australia	To evaluate the effectiveness of a specialty clinical pharmacist working in an ambulatory stem cell transplant clinic	Prospective cohort study<br>HSCT ambulatory clinic of a single metropolitan Australian tertiary hospital<br>12-months	N = 23<br>47 (18-63) years<br>Female = 13 (56.6%)<br>NR<br>NR<br>NR<br>NR	NR
Polito et al., 2021 [25], Canada	To assess:<br>1. the impact of a SMP on patient medication knowledge, self-efficacy, adherence, and to determine whether the impact was maintained over time<br>2. patient safety during implementation of the SMP<br>3. the acceptability of the SMP by assessing patient and staff perceptions and satisfaction with the program	Prospective pre- and post-cohort comparison study<br>Allo-HSCT units at the Princess Margaret Cancer Center<br>December 2017-August 2018	N = 51<br>N = 25 (49.0%) (intervention group, SMP)<br>N = 26 (51.0%) (comparison group)<br>< 65 years old = 19 (76%) (intervention group)<br>< 65 years old = 18 (69%) (comparison group)<br>Male = 18 (72%) (intervention group)<br>Male = 18 (69%) (comparison group)<br>NR<br>Acute leukaemia = 14 (56%), other = 11 (44.0%) (intervention group)<br>Acute leukaemia = 17 (65%), other = 9 (35.0%) (comparison group)<br>NR<br>NR<br>3-5 weeks after discharge	NR
Zanetti et al., 2022 [26], Brazil	To evaluate the results of pharmacotherapeutic follow-up on medication compliance and on the	Single-arm, open-label and non-randomized intervention study	N = 27<br>N = 18 (66.7%) adults<br>N = 9 (33.3%) children	The article refers to the term compliance and not adherence

	knowledge about pharmacotherapy of patients who underwent allo-HSCT	Allo-HSCT outpatient clinic in the Clinical Hospital of the Ribeirão Preto Medical School, University of São Paulo<br>May 2017-April 2018	N = 27<br>29.3 ± 18.9 (7-60) years<br>N = 27<br>Male = 15 (55.6%)<br>NR<br>N = 27<br>ALL = 10 (37.0%)<br>AML = 9 (33.3%)<br>Other = 8 (29.7%)<br>N = 27<br>HLA-identical = 11 (40.7%)<br>MUD = 8 (29.6%)<br>Haploidentical = 8 (29.6%)<br>Until 100 days	
		**Correlational study**		
Hoodin, 1993 [10], USA	1. To examine the rate of NA (MNA, personal-hygiene and environmental restrictions NA)<br>2. To examine the behavioural and psychological predictors of NA	Prospective correlational study from a dissertation thesis<br>HSCT program at Detroit Medical Centre<br>NR	N = 56*<br>38.1 ± 11.1 (18-60) years<br>Male = 32 (57.1%)<br>Caucasian = 50 (89.3%)<br>African-American = 4 (7.1%)<br>Hispanic = 2 (3.6%)<br>AML = 13 (23.2%)<br>CML = 13 (23.2%)<br>NHL = 10 (17.9%)<br>LH = 7 (12.5%)<br>MDS = 6 (10.7%)<br>Other = 13 (23.2%)<br>Allogeneic = 41 (73.2%)<br>Autologous = 15 (26.8%)<br>84-100 days	NR<br>Adherence refers to medication use, personal-hygiene and environmental restrictions
		**Cohort studies**		
Scherer et al., 2021 [15], Germany	1. To explore the predictive value of the pretransplant psychosocial screening tool TERS for overall survival<br>2. To assess patients' attitudes to immunosuppressive therapy using	Prospective study<br>Department I of Internal Medicine of the University Hospital of Cologne<br>May 2012-September 2017	N = 61<br>52.6 ± 15.4 (19-76) years<br>Male = 38 (62.3%)<br>NR<br>AML = 28 (45.9%)<br>NHL = 18 (29.5%)<br>ALL = 5 (8.2%)	NR

	the MESI, as a proxy parameter for MA		Other = 10 (16.4%)<br>NR<br>From 3 months to 3 years	
Posluszny et al.,<br>2022 [9],<br>USA	To prospectively investigate rates and predictors of NA to components of the post-HSCT medical regimen during the first 8 weeks after hospital discharge	Longitudinal prospective study<br>University of Pittsburgh Medical Center-Hillman Cancer Center<br>NR	N = 183<br>N = 92 patients<br>N = 91 caregivers<br>53.9 ± 9.9 (22-68) years (patients)<br>52.2 ± 13.7 (18-86) years (caregivers)<br>Male = 50 (54.0%) (patients)<br>Female = 27 (70.0%) (caregivers)<br>White race = 87 (95.0%)<br>AML = 55 (60.0%)<br>MDS = 14 (15.0%)<br>Other = 23 (25.0%)<br>MUD = 48 (52.0%)<br>HLA-identical = 34 (37.0%)<br>Haploidentical = 10 (11%)<br>NR<br>From 4-14 days prior HSCT to 8 weeks after discharge	NR<br>Adherence includes the following categories: medication use, infection precautions, lifestyle, central venous access management, alcohol and tobacco consumption and clinic appointments
**Studies reporting prevalence data**				
García-Basas et al.,<br>2020 [8],<br>Spain	To measure:<br>1. adherence to CsA, FK and sirolimus against secondary graft failure<br>2. adherence to CsA, FK, sirolimus and mycophenolate against GvHD<br>3. posaconazole, voriconazole, valganciclovir prophylaxis against infections<br>4. incidence of acute complications and readmission rates based on adherence	Retrospective observational study<br>Third-level Spanish hospital<br>May 2017-May 2018	N = 46<br>47.7 ± 12.1 years<br>Male = 32 (69.6%)<br>NR<br>AML = 14 (30.4%)<br>MDS = 8 (17.4%)<br>NHL = 4 (8.7%)<br>Other = 20 (43.5%)<br>Haploidentical = 15 (32.6%)<br>HLA-identical = 14 (30.4%)<br>MUD = 13 (28.3%)<br>100 days post engraftment	MA is the amount of medication a patient actually takes as compared with the amount prescribed by their physician
Lehrer et al.,<br>2018 [32],<br>France	To assess MA among allo-SCT recipients	Pilot observational study<br>Haematology Department of Saint-Antoine Hospital in Paris<br>November 2015-July 2016	N = 33<br>54 (31-70) years<br>Male = 20 (60.6%)<br>NR	NR

	AML = 16 (48.5%)<br>ALL = 7 (21.2%)<br>Other = 10 (30.3%)
	NR
	Mean 72 days

aGvHD: acute graft-versus-host disease; ALL: acute lymphoblastic leukaemia; allo-HSCT: allogeneic-hematopoietic stem cell transplant; AML: acute myeloid leukaemia; cGvHD: chronic graft-versus-host disease; CLL: chronic lymphocytic leukaemia; CML: chronic myeloid leukaemia; CsA: cyclosporine A; FK: tacrolimus; GvHD: graft-versus-host disease; HL: Hodgkin's lymphoma; HLA: human-leukocyte antigen; HSCT: haematopoietic stem cell transplantation; MA: medication adherence; MDS: myelodysplastic syndrome; MESI: Medication Experience Scale for Immunosuppressants; MM: multiple myeloma; MNA: medication non-adherence; MPS: myeloproliferative syndrome; MUD: matched unrelated donor; N: number; NA: non-adherence; NHL: non-Hodgkin's lymphoma; NR: nor reported; SD: standard deviation; SIPAT: Stanford Integrated Psychosocial Assessment for Transplantation; SMP: self-medication program; TERS: Transplant Evaluation Rating Scale.

*including both autologous and allogeneic HSCT
